# Supplementation of fetal bovine serum increased the quality of *in vitro* fertilized porcine embryo

**DOI:** 10.5455/javar.2021.h549

**Published:** 2021-11-01

**Authors:** Dibyendu Biswas, Sang Hwan Hyun

**Affiliations:** 1 Laboratory of Veterinary Embryology and Biotechnology, College of Veterinary Medicine, Chungbuk National University, Chungbuk 28644, Republic of Korea; 2 Department of Medicine, Surgery and Obstetrics, Faculty of Animal Science and Veterinary Medicine, Patuakhali Science and Technology University, Barisal campus, Bangladesh

**Keywords:** FBS, BCL-2, ROS, PZM-3

## Abstract

**Objective:**

The present study aimed to explain the effect of fetal bovine serum (FBS) on the *in vitro* production of porcine embryos and the molecular effects of FBS on the growing of porcine embryos.

**Materials and Methods:**

Immature porcine oocytes were matured and fertilized *in vitro*. The resulting zygotes were cultured in porcine zygotic medium-3- until day 7 and FBS was added on day 4. Without FBS, it was treated as a control group. Quantitative real-time PCR and 2′,7′-dichloro-dihydro-fluorescein diacetate (H_2_DCFDA) molecular staining techniques were used to detect the expression patterns of apoptosis-associated genes and the accumulation of reactive oxygen species (ROS), respectively. Paired student’s *t*-test was used by GraphPad Prism statistical software.

**Results:**

FBS supplementation boosted blastocyst (BL) development and total cell count per BL substantially (*p* < 0.05). However, hatching and hatched BLs also increased in the FBS-treated group compared to the control. We also found that ROS accumulation in FBS-treated embryos was significantly reduced (*p* < 0.05) compared to the control group. The expression of the anti-apoptotic gene BCL-2 was significantly increased in FBS-treated BLs, but the pro-apoptotic gene, caspase-3 expression, was significantly reduced in FBS-treated BLs.

**Conclusion:**

Our results suggest that FBS supplementation in porcine culture media could increase porcine embryo production by decreasing ROS accumulation and increasing the anti-apoptotic gene expression in developing BLs.

## Introduction

*In vitro* porcine embryo production is essential for biomedical research as pigs are used for xenotransplantation research concerning the new drug discovery process [1]. However, unlike *in vivo* embryo production, *in vitro* high-quality embryo production is very challenging. To beat this challenge, many attempts were introduced to develop gold standard protocols for embryo production. Supplementing different growth factors/hormones or other nutrients during the developmental stage can reduce the stress caused by *in vitro* conditions [2,3]. During *in vitro* embryo production, some byproducts are generated inside embryos that are detrimental to their development. However, they are very unstable and highly reactive with other molecules that also donate or accept an electron. These free radicals include nitric oxide, superoxide anion, hydrogen peroxide, oxygen singlet, hydroxyl, hypochlorite, and peroxynitrite [4]. These free radicals mostly target the vulnerable macromolecules and cause cell damage, thus disrupting the embryo’s homeostasis environment.

Several research groups have developed different embryo culture media, such as North Carolina State University (NCSU) 23 and 37 media [5], Iowa State University medium [6], and porcine zygotic medium-3 (PZM-3) [7], to be studied for *in vitro* culture (IVC) of porcine embryos. However, NCSU23 and PZM-3 media have been widely used to culture porcine embryos. Porcine zygotes of various origins, either *in vitro* fertilization (IVF), parthenogenesis, or somatic cell nuclear transfer, have been reported to have higher development rates in PZM-3 media than in NCSU23 media [8]. It was also reported that the embryonic development rate was increased when the medium was supplemented with fetal bovine serum (FBS) [9] or fetal calf serum (FCS) [10]. It was proved that supplementation of animal serum acts as an energy source, osmoregulator, pH stabilizers, numerous antioxidants, and growth factors [11,12]. 

During the early growing phase, the embryo needs more energy due to the presence of more blastomeres in the embryo. Embryo development and hatching rates were increased when FBS or FCS was integrated into the embryo culture media during the mouse and rat model experiments [13]. However, the mechanism underlying how the integration of FBS into IVC medium enhances embryonic development in the laboratory is still unknown. On the other hand, during *in vitro *embryo culture, blockage of four to eight cells is a common phenomenon [14]. Many approaches have been adopted to overcome this obstacle by supplementing with hypotaurine, FBS, FCS, and bovine serum albumin (BSA) during IVC of* the *porcine embryo. Integrating serum into the embryo culture medium can improve embryo quality by preventing apoptosis and/or decreasing reactive oxygen species (ROS) accumulation. Therefore, this study was designed to investigate the role and the molecular mechanisms of porcine embryo development during the integration of FBS into culture media.

## Materials and Methods

### Chemicals

All the chemicals used in this experiment were bought from Sigma-Aldrich, St. Louis, MO, unless otherwise stated.

### Ovary collection, recovery, and in vitro oocyte maturation 

Pre-pubertal gilt ovaries were collected from an abattoir house in Chungbuk city, and the collected ovaries were transported to the laboratory within 2 h. The transport medium consisted of NaCl (0.9%), streptomycin sulfate (100 mg/ml), and penicillin-G (100 IU/ml). The contents of the follicular (3–7 mm in diameter) were aspirated with a 5 ml disposable syringe with a 20 gauge needle. A minimum of three layers of compact cumulus cells with homogenous ooplasmic cumulus–oocyte complexes (COC) was selected using a stereomicroscope. A selected group of 50–60 immature COCs were placed in 4-well culture dishes (Nunc, Roskilde, Denmark) with the oocyte maturation medium. The media consisted of tissue culture medium 199 (Invitrogen, Grand Island, NY), cysteine (0.6 mM), pyruvate (0.91 mM), epidermal growth factor (15 ng/ml), kanamycin (75 μg/ml), insulin (1 μg/ml), and porcine follicular fluid (pFF) (10%, v/v). A four-well dish containing immature COCs was then placed at 39°C in an exceedingly humidified atmosphere containing 5% CO_2_. These culture conditions were carried out for 20–22 h with 10 IU/ml equine chorionic gonadotropin (eCG; Intervet International BV, Boxmeer, Netherlands) and 10 IU/ml human chorionic gonadotropin (hCG; Intervet International BV, Boxmeer, Netherlands). After 20–22 h of culture, the oocytes were rinsed with fresh medium and cultured again for an additional 18 h without hormonal supplementation. The pFF was prepared according to Biswas et al. [15] and preserved at −20°C for further use. For maturation, each well of the 4-well Nunc plate contained 500 μl of maturation medium.

### IVF of porcine oocytes and culture

IVF and IVC were carried out according to our previous study [16]. Briefly, on day 2, after *in vitro* maturation (IVM), de-cumulated M-II oocytes were co-incubated for 20 min with spermatozoa at a concentration of 5 × 10^5^/ml in 50 μl microdrop of modified Tris buffer medium (mTBM) at 39°C in a humidified atmosphere containing 5% CO_2_ and 95% air. After 20 min of co-incubation, gentle pipetting was applied to remove loosely attached sperms from the zona pellucida. The zygotes were then washed thrice with mTBM and incubated with mTBM without sperm for 5–6 h in the same environment. After that, the zygotes were washed thrice with PZM-3 medium and cultured in 30 μl microdrops (10 zygotes per drop) of PZM-3 and covered with prewarmed mineral oil. The putative zygotes were incubated at 39°C for 168 h in a humidified atmosphere containing 5% O_2_, 5% CO_2_, and 90% N_2_. The zygotes were randomly allocated into two groups after successful IVF. On day 4 post-IVF, 10% FBS was injected into the culture medium, and without FBS was treated as the control group. Each experiment was repeated at least thrice.

### Embryo evaluation and total blastocyst (BL) count

On day 2 and day 7, zygotes were checked for cleavage and BL formation, respectively. To calculate the total number of blastomere in the BL at day 7, the BL was washed with 1% (wt/vol) phosphate-buffered saline BSA (PBS-BSA) for 5 min and stained with 5 mg/ml of Hoechst 33342 (bisbenzimide). Subsequently, the embryos were briefly fixed in 4% paraformaldehyde. Next, the BL was mounted on a glass slide in a drop of 100% glycerol and gently covered with a cover slip. The stained BLs were observed at 400x using a fluorescence microscope (Nikon Corporation, Japan) and counted the total number of blastomere.

### Quantitative real-time reverse transcription polymerase chain reaction (RT-PCR) and the gene expression pattern

In each group, approximately 8–10 IVF BLs were stored at −80°C after washing with PBS until further use for the gene expression analysis. Quantitative real-time RT-PCR analyzed the expression of B cell lymphoma-2 (BCL-2) and caspase-3 mRNA in BL. TRIzol reagent (Invitrogen, Carlsbad, CA) was used to extract the total RNA from polled BLs according to the manufacturer’s instructions. A spectrophotometer (Micro UV-Vis spectrophotometer, Hangzhou, 310023, China) was used to measure the total RNA concentration at a wavelength of 260 nm. A mixture of moloney murine leukemia virus reverse transcriptase (Invitrogen, Carlsbad), random primer (9-mers; TaKaRa Bio, Otsu, Shiga, Japan), and 1 μg of total RNA was used to synthesize the first-strand complementary DNA (cDNA). Real-time RT-PCR was carried out in a 20 μl PCR reaction volume consisting of 10 μl of 2 × SYBR premix Ex Taq (TaKaRa Bio Inc.), 1 μl cDNA, 1U of Taq polymerase (Intron Bio Technologies, Seongnam, Korea), and 10 pM of each specific primer in an MX3000P thermocycler engine (Stratagene-Agilent Technologies, Waldbronn, Germany). 

The reaction was set for 40 cycles with the following parameters: denaturation at 95°C for 30 sec, followed by 30 sec of annealing at 57°C and 30 sec extension at 72°C. All oligonucleotide primer sequences are presented in Table 1. At the end of the extension, the fluorescence intensity of each gene was recorded from the PCR product. The fluorescence intensity threshold value of all samples was set manually. The number of reaction cycles where the fluorescence intensity of each PCR product crossed the threshold value was considered the cycle threshold (Ct) in the exponential phase of PCR amplification. The relative comparative quantification of the expression of each target gene was based on the Ct values at constant fluorescence intensity. It was normalized to the RN18S as an internal control gene. The relative mRNA expression of each target gene (*R*) was calculated using the following equation, *R* = 2-[ΔCt sample-ΔCt control]. The experiment was repeated at least thrice.

### ROS accumulation assay in BLs 

On day 7, BLs from both groups were used for intracellular ROS analysis. Briefly, the BLs were incubated with 10 mM of 2′,7′-dichloro-dihydro-fluorescein diacetate (H_2_DCFDA; Invitrogen) for 30 min in a dark place, and after incubation, the treated BLs were washed thrice in HEPES-buffered Tyrode’s medium-polyvinyl alcohol (TLH-PVA) solution. The stained BLs were mounted on a glass slide and visualized under an epifluorescence microscope (TE2000-U, Nikon, Japan). The final photos were captured with a digital camera and the fluorescence intensity of both BLs was measured with the ImageJ software (version 1.47; National Institutes of Health, USA). The fluorescence intensity was normalized with the control. This experiment was repeated at least four times.

### Statistical analysis

The data percentage of the cleaved embryo and various types of BL formation was analyzed by paired student’s *t*-test using GraphPad Prism software. All data are presented as mean ± SEM. Differences at *p* < 0.05 were considered statistically significant

**Table 1.** Sequences of primer sets used for selective gene expression on day 7 porcine BL.

Symbol	Primer sequences (5′-3′)	Product size	Accession number
BCL-2	F: AGGGCATTCAGTGACCTGAC	193	NM_214285
	R: CGATCCGACTCACCAATACC		
Caspase-3	F: CGTGCTTCTAAGCCATGGTG	186	NM_214131
	R: GTCCCACTGTCCGTCTCAAT		
RN18S	F: CGCGGTTCTATTTTGTTGGT	219	NR_046261.1
	R: AGTCGGCATCGTTTATGGTC		

**Table 2.** Effects of FBS supplementation on *in vitro* development of porcine IVF zygote.

Culture system	Total zygotes culture	Cleavage (%)	BL (%)	Average blastomere/BL
Control	573	310 (55.58 ± 8.05)	138 (23.85 ± 3.04) ^a^	46.20 ± 0.84^a^
+FBS	901	606 (68.19 ± 4.50)	353 (38.23 ± 3.98) ^b^	102.2 ± 1.50^b^

^a,b^ Values with different superscripts in the same column are significantly different (*p* < 0.05).

Data are given as mean ± SEM from each replicate.

**Figure 1.**
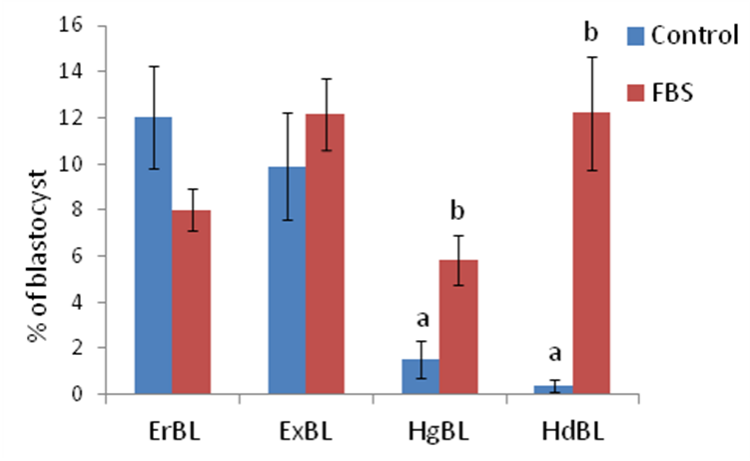
Effect of FBS on *in vitro* production of porcine embryo quality on day 7. The data are presented as mean ± SEM. Bars with different letters in each endpoint differ from each other, which are statistically significant (*p* < 0.05). ErBL: early blastocyst; ExBL: expanded blastocyst; HgBL: hatching blastocyst; HdBL: hatched blastocyst.

## Results

### Effects of FBS on BL quality derived from IFV embryos in PZM-3 culture medium

**Figure 2.**
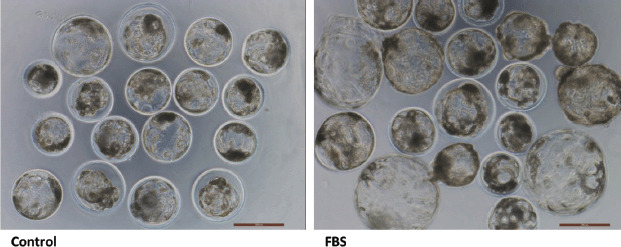
On day 7, the different types of BL were found with or without FBS supplementation during *in vitro* porcine embryo production. Bar = 100 mm.

As shown in Table 2, the embryo developmental potential at the BL stage and the total blastomere per BL were significantly higher (*p* < 0.05) in the FBS supplemented group than in the control group. On day 2, the total cleavage rate was not significantly different between the two groups. Furthermore, the group that received FBS during the culture period also showed significant (*p* < 0.05) hatching rates (5.82 ± 1.08) and hatched BL formation (12.22 ± 2.47) compared to the control group (1.51 ± 0.81and 0.4 ± 0.26, respectively). However, there was no significant difference in early BL and expanded BL formation between the groups (Fig. 1). Morphologically, embryos appeared to look good in the FBS-treated group compared to the control group (Fig. 2).

### Effect of FBS on ROS production in day-7 porcine BLs

**Figure 3.**
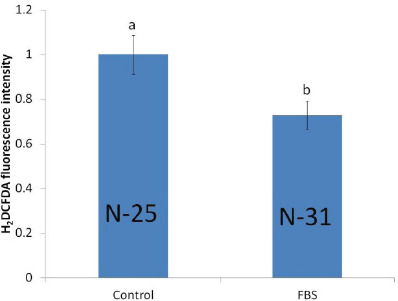
The fluorescence intensity of ROS levels was detected by H_2_DCFDA staining of BL derived from IVF embryos cultured with FBS in the PZM-3 culture medium. The fluorescence intensity of BLs was analyzed by ImageJ software. The arbitrary data according to the control group was set as 1. The number of BLs examined in each group is shown in the bars. Values represent mean ± SEM of the separate experiments. Bars with different letters in their respective endpoints differ from each other and are treated as statistically significant (*p *< 0.05).

The H_2_DCFDA molecular staining technique was applied to the determination of ROS levels on day-7 BLs. The present study showed that the ROS production was significantly reduced (*p* < 0.05) in BL cultured with FBS compared to the untreated control group (lower H_2_DCFDA signal) (Fig. 3).

### Effect of FBS supplementation on the mRNA expression of apoptosis-related genes in day-7 porcine BLs

**Figure 4.**
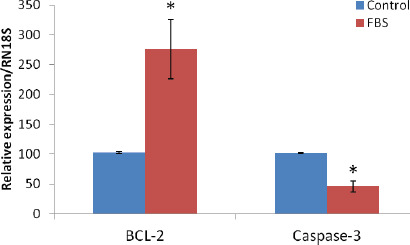
Relative levels of BCL 2 and caspase 3 mRNA expression IVF-derived porcine BLs cultured with FBS and without FBS (control group) in the PZM-3 culture medium. The experiment was repeated at least thrice. The data are expressed as mean ± SEM of this experiment (*p <0.05).

The relative abundance of BCL-2 mRNA in day-7 BL was significantly higher (*p* < 0.05) in FBS-treated BLs compared to control, and caspase-3 mRNA level in day-7 BL was also significantly lower (*p* < 0.05) in the FBS treatment group compared to the control group (Fig. 4).

## Discussion

FBS is the most widely used cell culture and can also be used as an energy source for growing cells [17]. It supplies cocktail factors for cell growth, proliferation, and attachment [18]. Several studies have shown that supplementation of serum-like FBS in IVM or IVC medium increased the development of the embryo other than BSA or other macromolecules. In the present study, 10% FBS was supplemented on day 4 in the PZM-3 during the culture of *in vitro* fertilized porcine embryos. The percentages of BLs development and the total cell number per BL were significantly increased in the medium supplemented with FBS. Similar results were found in IFV porcine embryo development where 10% FBS was added in the chemically defined medium [19], and also 2.5% FBS was used for IVF bovine embryo production [20]. However, it was found that 5% FBS has been advantageous during the pre-cleavage stage, and 10%–20% FBS has been advantageous during the post-cleavage stage of the bovine embryos and also overcomes the deleterious effects of serum on early developing embryos by exploiting embryotrophic effects during later stage [21]. 

Another study on bovine embryo culture indicated that adding FBS in the culture medium promoted development to the morula and BL stage [20]. Mostly, FBS acts as a protein source in the culture medium. Numerous embryonic growth-promoting factors and other unknown cell survival components are essential during embryonic growth [22]. Adding FBS on day 4 may improve the metabolic pathways when the IVF embryos are mostly at the morula stage [23], and at that time, embryos need more nutrients and energy. It has also been reported that serum strongly inhibits the division of the first stage of the fertilized embryos and has no beneficial effect on the development from two cells to the morula stage [24]. This study found that the total cell number per BL on day 7 was increased twice when FBS was introduced into the embryo culture medium on day 4. A growing embryo needs more energy for replicating quickly and smoothly. The presence of FBS in the culture medium influences the embryonic cell division in the BL due to increasing intracellular lipid metabolism [23,25]. 

The increased metabolism leads to the formation of new cells in growing embryos. It could be suggested that the presence of one of the main factors in FBS is phosphate, and the concentration is higher as inorganic phosphorus can stimulate the development of eight-cell embryos in a rat model [26]. Moreover, it was also found that adding FBS on day 4 also improved the hatching and hatched BL formation compared to the control. It indicated that FBS had biphasic effects on developing embryos [24], but the stimulation from the serum at late-stage embryo development is unknown. However, FBS could trigger the cell cycle process and result in more BL and blastomere found in this experiment. Indeed, increased total blastomere dictating that developmental competence was excellent in accordance with trophectoderm cell number [27], and the total cell number is the suitable paramere for assessing *in vitro* produced embryos.

Our study found that with H_2_DCFDA staining of day-7 BLs, ROS generation was significantly decreased in FBS-treated embryos. Several other studies have found that adding FBS to the IVC media often triggers ROS accumulation in mammalian cells [28]. In that case, bovine embryos were used and nicotinamide adenine dinucleotide phosphate (NADPH) oxidases enzymes were activated in the presence of FBS, which led to accelerating the cell cycle progression [29]. But this study showed the opposite result where ROS generation was decreased in the presence of FBS in culture medium. The variation of this result may be due to the different types of embryos used in this study. The porcine embryo contains high lipid contents in the ooplasm and has different metabolic activities during embryogenesis. Another study indicated that numerous antioxidant transcripts were upregulated after adding FBS in the culture medium. Excessive accumulation of ROS can damage the cellular component of growing embryos, and FBS inhibits the ROS production by activation of p-p38 mitogen-activated protein kinase (MAPK) and phospho-a serine/threonine protein kinase (p-AKT) signal pathways [19]. 

Programmed cell death or apoptosis is jointly involved in early embryonic development and differentiation. An unfavorable culture media sometimes induces unscheduled embryonic cells’ apoptosis, leading to cessation or abnormal early developmental processes [30]. However, the balanced expression of pro- and anti-apoptotic genes controls the apoptotic pathway. The BCL-2 gene family is known for its pro- and anti-apoptotic subgroups, and the BCL-2 gene is known to protect against apoptosis. In this observation, it was found that the addition of FBS in the porcine embryo culture media significantly affected the anti-apoptotic gene BCL-2 in FBS-treated embryos and significantly decreased the expression of the pro-apoptotic gene, caspase-3 compared to the control group. Another subgroup is highly conserved to the regulator of apoptosis gene expression like caspase-3. From these observations, it can be explained that FBS can reduce the apoptotic process in porcine BLs by preventing apoptosis either by preventing the activation of caspase-3 or by preventing the release of factors that induce apoptosis [31]. 

BCL-2 is a potent suppressor of cell death and its upstream expression can reduce the gene that promotes cell death, such as caspase-3 in maternal stores in developing embryos. This would be consistent with the idea that superior quality embryos contain higher mRNA levels from the anti-apoptotic gene and conversely lower levels of the pro-apoptotic transcriptome. However, FBS had some detrimental effects when used in porcine parthenote, and in that case, the total numbers of cells per BL were significantly reduced and apoptotic cells were significantly increased [20]. In fact, increased expression of the anti-apoptotic gene BCL-2 in embryos determined the presence of embryos with good morphological quality in IVC media [32].

## Conclusion

In conclusion, these data indicated that the supplementation of the porcine culture media with FBS positively affects *in vitro* embryo development. The addition of FBS to the culture medium on day 4 significantly increased the hatched BL production *in vitro *and decreased ROS accumulation. Expression of the anti-apoptotic BCL-2 gene was significantly increased in developing BLs.

## List of Abbreviations

FBS, fetal bovine serum; PZM-3, porcine zygotic medium-3; ROS, reactive oxygen species; BL, blastocyst; NCSU, North Carolina State University; IVF, *in vitro* fertilization; FCS, fetal calf serum; COC, cumulus–oocyte complexes; pFF, porcine follicular fluid; IVC, *in vitro* culture; IVM, *in vitro* maturation; mTBM, modified Tris buffer medium; h, hours; min, minutes; sec, seconds; cDNA, complementary DNA; BCL-2, B cell lymphoma-2; eCG, equine chorionic gonadotropin; hCG, human chorionic gonadotropin; RT-PCR, reverse transcription polymerase chain reaction; TLH-PVA, HEPES-buffered Tyrode’s medium-polyvinyl alcohol; NADPH, nicotinamide adenine dinucleotide phosphate; MAPK, mitogen- activated protein kinase; p-AKT, phosphoa serine/threonine protein kinase.
